# New-Generation BeiDou (BDS-3) Experimental Satellite Precise Orbit Determination with an Improved Cycle-Slip Detection and Repair Algorithm

**DOI:** 10.3390/s18051402

**Published:** 2018-05-02

**Authors:** Chao Hu, Qianxin Wang, Zhongyuan Wang, Alberto Hernández Moraleda

**Affiliations:** 1 NASG Key Laboratory of Land Environment and Disaster Monitoring, China University of Mining and Technology, Xuzhou 221116, China; wqx@cumt.edu.cn; 2 School of Environment Science and Spatial Informatics, China University of Mining and Technology, Xuzhou 221116, China; Ahm_phd@yahoo.es; 3 Satellite Positioning for Atmosphere, Climate and Environment (SPACE) Research Centre, School of Science, Mathematical and Geospatial Sciences, RMIT University, Melbourne, VIC 3001, Australia

**Keywords:** BDS-3 experimental satellites, precise orbit determination, improved cycle-slip detection and repair algorithm, improved methods, normal equation stacking, step-by-step

## Abstract

Currently, five new-generation BeiDou (BDS-3) experimental satellites are working in orbit and broadcast B1I, B3I, and other new signals. Precise satellite orbit determination of the BDS-3 is essential for the future global services of the BeiDou system. However, BDS-3 experimental satellites are mainly tracked by the international GNSS Monitoring and Assessment Service (iGMAS) network. Under the current constraints of the limited data sources and poor data quality of iGMAS, this study proposes an improved cycle-slip detection and repair algorithm, which is based on a polynomial prediction of ionospheric delays. The improved algorithm takes the correlation of ionospheric delays into consideration to accurately estimate and repair cycle slips in the iGMAS data. Moreover, two methods of BDS-3 experimental satellite orbit determination, namely, normal equation stacking (NES) and step-by-step (SS), are designed to strengthen orbit estimations and to make full use of the BeiDou observations in different tracking networks. In addition, a method to improve computational efficiency based on a matrix eigenvalue decomposition algorithm is derived in the NES. Then, one-year of BDS-3 experimental satellite precise orbit determinations were conducted based on iGMAS and Multi-GNSS Experiment (MGEX) networks. Furthermore, the orbit accuracies were analyzed from the discrepancy of overlapping arcs and satellite laser range (SLR) residuals. The results showed that the average three-dimensional root-mean-square error (3D RMS) of one-day overlapping arcs for BDS-3 experimental satellites (C31, C32, C33, and C34) acquired by NES and SS are 31.0, 36.0, 40.3, and 50.1 cm, and 34.6, 39.4, 43.4, and 55.5 cm, respectively; the RMS of SLR residuals are 55.1, 49.6, 61.5, and 70.9 cm and 60.5, 53.6, 65.8, and 73.9 cm, respectively. Finally, one month of observations were used in four schemes of BDS-3 experimental satellite orbit determination to further investigate the reliability and advantages of the improved methods. It was suggested that the scheme with improved cycle-slip detection and repair algorithm based on NES was optimal, which improved the accuracy of BDS-3 experimental satellite orbits by 34.07%, 41.05%, 72.29%, and 74.33%, respectively, compared with the widely-used strategy. Therefore, improved methods for the BDS-3 experimental satellites proposed in this study are very beneficial for the determination of new-generation BeiDou satellite precise orbits.

## 1. Introduction

The BeiDou demonstration system (BDS-1), the regional service system (BDS-2), and the global service system (BDS-3) have been developed by a “three-step” strategy [1]. BDS-1 consists of geosynchronous orbit (GEO) satellites launched from 2000 to 2003. On 27 December 2012, the BeiDou system, which has a space constellation of five GEO, five inclined geosynchronous orbit (IGSO) satellites, and four medium Earth orbit (MEO) satellites, began to provide services to the Asia Pacific region. BeiDou started to evolve from a regional service capability to a global service capability with the launch of the new-generation BeiDou experimental satellite (BeiDou, I1-S) into orbit in March 2015. Five BDS-3 experimental satellites (C31–C35) and two BDS-3 satellites (C19 and C20) were in orbit by the end of November 2017 [2]. Moreover, the new-generation BeiDou system plans to achieve a 30 satellites network by 2020 (three GEO, 24 MEO, and three IGSO) providing global navigation, positioning, and timing services [3].

However, the orbit accuracy of BeiDou is currently one of the major challenges with the expanding application of BeiDou in the field of navigation and positioning. Researchers have assessed the BDS-2 orbit [4,5,6] well to improve the accuracy and relevant algorithms of BeiDou orbit determination. The results showed that the three-dimensional root-mean-square error (3D RMS) of BDS-2 one-day overlapping arc for MEO (and IGSO) and GEO were improved from 0.5 m and 3.0 m to 0.2 m and 1.0 m, respectively. Furthermore, the orbit models of BDS-2 were refined in recent studies, which included the introduction of a radial constant acceleration of GEO [7,8], yaw attitude model [9,10], hardware delay [11], and inter-frequency bias (IFB) [12] to further improve the orbit accuracy. Recent assessment studies suggested that the satellite laser range (SLR) residuals from different data analysis centers for BDS-2 orbits are better than 0.2 m for MEO (and IGSO) and 0.5 m for GEO [11], respectively. In general, BDS-2 orbits have been improved and refined over the years. For the new-generation BeiDou experimental satellites (hereafter called BDS-3) orbits, in [13,14], four BDS-3 orbits (C31–C34) were estimated based on the international GNSS Monitoring and Assessment System (iGMAS) and the Multi-GNSS Experiment (MGEX) stations. Due to restrictions in the tracking networks, the results suggested that the radial and along-directions of one-day overlapping arc errors were between 0.10 m and 0.25 m. In addition, the RMS of the SLR residuals was at the 0.1–0.3 m level. However, the impacts of orbit models, such as solar radiation pressure and yaw model, were not analyzed in their studies. In [15], three-month BDS-3 orbits were acquired based on the same methods as BDS-2 orbit determination, which was analyzed in terms of yaw attitude and solar radiation pressure models. Moreover, to further improve BDS-3 orbit accuracy, inter-satellite link (ISL) and autonomous orbit determination were taken into account [16,17,18]. However, compared with the BDS-2 orbit, only preliminary studies on BDS-3 orbit determination, which should be focused on, were conducted.

To improve the accuracy of BDS-3 orbits, it should be noted that the data availability and the quality of tracking networks used are the two main factors that restrain the orbit accuracy in the BDS-3 orbit determination, which directly affect the accuracy of orbit determination [19]. Therefore, in [20,21,22], the BDS-3 signals and its quality were evaluated, and the results, compared to BDS-2, showed that the data quality of the BDS-3 improves significantly. In addition, the satellite-induced multipath effects along with the elevation in BDS-2 disappeared in the BDS-3 observations. However, because the new signals of BDS-3 satellites remain in the internal test stage, the BDS-3 signals are mainly tracked by the iGMAS network [13], the data quality of which was not involved in the related analysis. Meanwhile, from the experiments of data quality analysis (Section 2), it was found that the smaller cycle-slip ratio, especially for GPS, in the iGMAS tracking data was apparent. Thus, research on the optimal iGMAS observations is vital for the BDS-3 orbit determination.

To control the GNSS observation quality, the effective cycle-slip detection and repair algorithm plays a key role [23]. When triple-frequency observations of BDS and GPS are received, the linear combinations of triple-frequencies make it easy to realize cycle-slip detection and repair in each frequency [24]. However, most of stations for tracking BDS-3 signals are still double frequencies. In [25], the Turboedit algorithm was proposed, which has been widely used in the field of navigation and positioning. However, it should be noted that the observation noise and poor data quality might impose restrictions on this algorithm, especially for omitting the observations with several cycle slips that reduces the data availability of the network directly. Therefore, an accurate cycle-slip detection and repair algorithm in BDS-3 orbit determination should be proposed to improve data availability given the limited data sources and the poor quality of iGMAS data.

Furthermore, to increase the data availability, the estimation of parameters based on the normal equation stacking (NES) of several networks is another method, thereby improving the parameter’s strength in BDS-3 orbit determination. Since the iGMAS network can receive BDS-3 signals (B1 and B3) with a few stations but not consistent with BDS-2 signals (mainly B1 and B2) tracked by the MGEX network. The relevant studies about the NES were discussed based on the single-day into multi-day solutions [26,27,28]. These served as references for improving the parameter estimation in BDS-3 orbit determination in this study.

To determine four new-generation BeiDou (BDS-3) experimental satellites (C31, C32, C33, C34; C35 could not be tracked by iGMAS) orbits with the given limited data availability and poor data quality of iGMAS data, this study mainly proposes an improved cycle-slip detection and repair algorithm and two optimized methods based on iGMAS and MGEX networks. In Section 2, the quality of iGMAS observations is briefly analyzed. Then, an improved cycle-slip detection and repair algorithm for iGMAS is proposed to solve the poor data quality and low data availability. In Section 3, two orbit determination methods are discussed based on the combination of MGEX and iGMAS observations. In Section 4, orbit accuracy analysis is conducted by overlapping arc errors and SLR residuals, respectively.

## 2. Improved Cycle-Slip Detection and Repair Algorithm

In the analysis data of sixteen iGMAS stations could be obtained by the end of December 2016, among them, nine stations tracked BDS-3 signals. To understand the data quality of iGMAS, two stations (LHA1 and WHU1) were selected to conduct data quality analysis. Meanwhile, it should be noted that the corresponding MGEX stations (LHAZ and JFNG) were the same location stations and can be used for data quality comparison. In the experiments, the GPS data from day of year (DOY) 156 to 160 in 2017 were selected as examples. The observation effective rate, MP1 and MP2, and the average cycle-slip ratio (CSR) (the ratio between the number of observations and epochs with cycle slips) [29] are listed in Table 1 based on the Multi-GNSS data analysis software (MTEQC), which is developed and improved by the authors. Moreover, the effective rate and CSR of BDS-3 in iGMAS data are also listed in Table 1, while the BDS-2 of MGEX was taken as a reference to compare BeiDou data quality of both networks.

In Table 1, the cut-off of elevation angle was set as 5 degrees. Analyses of observations taken at the same sites revealed that the data quality of GPS observations is considerably poorer in iGMAS than for MGEX, especially focusing on cycle slips and multipath. In addition, investigating BDS-2 and BDS-3 observations, the cycle slips are much more numerous in MGEX. However, the full exploitation of observations is necessary due to limited data availability in BDS-3 orbit determination. Thus, the high-accuracy cycle-slip detection and repair algorithm for iGMAS and MGEX is a prerequisite of the BDS-3 orbit determination.

To optimize the observations quality in BDS-3 orbit determination, based on the traditional Turboedit algorithm [25], the following issues may occur when processing the cycle slips in iGMAS data: (1) the detection and repair of the small cycle-slips is inaccurate given the larger noise of iGMAS data (MP1 and MP2 are larger than for MGEX in Table 1); (2) the data piece with several cycle slips is eliminated directly, thereby reducing the data availability of the tracking observations; and (3) the algorithm is insensitive to some special combinations (such as the same cycle slips in each frequency), especially small cycle slips. Therefore, an improved cycle-slip detection and repair algorithm for iGMAS data is presented in the study that is based on the accurate prediction of the ionospheric delays. The corresponding algorithm is presented below.

The GNSS observation equation is [30]:

(1) $$\left\{ \begin{array}{l} P_{i}=(\rho +c\cdot dT-c\cdot dt+d_{trop})+k_{i}\tilde{I}+\varepsilon_{i}\\ L_{i}=(\rho +c\cdot dT-c\cdot dt+d_{trop})-k_{i}\tilde{I}+\lambda_{i}N_{i}+\xi_{i} \end{array}\right.$$

Equation (1) shows the pseudo-range and phase observation equation; *i* denotes the carrier frequency; ρ is the geometric distance between the satellite and the station; *c* is the speed of light; dT,dt are the satellite and receiver clock offsets, respectively; $d_{trop}$ represents the troposphere delay; $\tilde{I}$ is the ionospheric delay on *L*_1_ frequency; $k_{i}=f_{1}^{2}/f_{i}^{2}$ is the ionospheric delay coefficient; $\varepsilon_{i},\xi_{i}$ are the corresponding observation noise; $\lambda_{i}$ is the wavelength; and $N_{i}$ is the integer ambiguity.

The ambiguity of Melborne-Wübbena (MW) combination reads:

(2) $$N_{w}=\left(\frac{f_{1}L_{1}-f_{2}L_{2}}{f_{1}-f_{2}}-\frac{f_{1}P_{1}+f_{2}P_{2}}{f_{1}+f_{2}}\right)\cdot \frac{1}{\lambda_{w}}=N_{1}-N_{2}+\varepsilon_{w}$$

where $\lambda_{w}=\frac{c}{f_{1}-f_{2}}$ is the widelane wavelength, and $\varepsilon_{w}$ is the combined noise.

The cycle slips of *L*_1_ and *L*_2_ are assumed as $\Delta N_{1},\Delta N_{2}$, respectively; thus, after determining the epoch difference of the MW combination, the combined ambiguity is:

(3) $$\Delta N_{w}=\Delta N_{1}-\Delta N_{2}+\varepsilon_{\Delta w}$$

where $\varepsilon_{\Delta w}$ is the noise. Then, Equation (3) is inserted into the phase equation as epoch difference:

(4) $$\begin{array}{l} \Delta L=\left[\begin{array}{l} \Delta L_{1}-\lambda_{1}\Delta N_{w}\\ \Delta L_{2} \end{array}\right]=\left[\begin{array}{cc} 1 & \lambda_{1}\\ 1 & \lambda_{2} \end{array}\right]\left[\begin{array}{l} \Delta (\rho +c\cdot dT-c\cdot dt+d_{trop})\\ \Delta N_{2} \end{array}\right]-\left[\begin{array}{l} k_{1}\\ k_{2} \end{array}\right]\Delta \tilde{I}+\left[\begin{array}{l} \Delta \xi_{1}\\ \Delta \xi_{2} \end{array}\right]\\ =A\left[\begin{array}{l} \Delta (\rho +c\cdot dT-c\cdot dt+d_{trop})\\ \Delta N_{2} \end{array}\right]-k\Delta \tilde{I}+\Delta \xi \end{array}$$

In Equation (4), $A=\left[\begin{array}{cc} 1 & \lambda_{1}\\ 1 & \lambda_{2} \end{array}\right]$, $k={\left[k_{1},k_{2}\right]}^{T}$, $\Delta \xi ={\left[\Delta \xi_{1},\Delta \xi_{2}\right]}^{T}$.

Assume $\rho_{0}=\rho +c\cdot dT-c\cdot dt+d_{trop}$. According to Equation (4), the cycle slips on *L*_2_ observation can be expressed as:

(5) $$\left[\begin{array}{l} \Delta {\hat{\rho }}_{0}\\ \Delta {\hat{N}}_{2} \end{array}\right]={\left(A^{T}Q^{-1}A\right)}^{-1}A^{T}Q^{-1}\Delta L$$

and:

(6) $$\left\{ \begin{array}{l} Q=\mathrm{cov}\left[-k\Delta \tilde{I}+\Delta \xi \right]=\sigma_{\Delta \tilde{I}}^{2}kk^{T}+Q_{\Delta \xi \Delta \xi }-kc^{T}-ck^{T}\\ c=\mathrm{cov}\left[\Delta \xi ,\Delta \tilde{I}\right] \end{array}\right.$$

It should be noted that the accurate estimation of the cycle-slip values on *L*_2_ requires further analysis of ***Q***. The parameter $\Delta \tilde{I}$ in Equation (4) can be expressed as the difference between the estimation error of the former epoch $d{\hat{I}}_{\left(t-1\right)}$ and the prediction error of the current epoch $d{\bar{I}}_{\left(t\right)}$:

(7) $$\Delta \tilde{I}=d{\bar{I}}_{\left(t\right)}-d{\hat{I}}_{\left(t-1\right)}+e_{m}$$

where $e_{m}$ is the prediction model error. However, for the epoch without cycle slips, the phase observation equation can be obtained as below:

(8) $$L=\left[\begin{array}{l} L_{1}-\lambda_{1}N_{1}-c\cdot dT+c\cdot dt-d_{trop}\\ L_{2}-\lambda_{2}N_{2}-c\cdot dT+c\cdot dt-d_{trop} \end{array}\right]=\left[\begin{array}{cc} 1 & -k_{1}\\ 1 & -k_{2} \end{array}\right]\left[\begin{array}{l} \rho \\ I \end{array}\right]+\left[\begin{array}{l} \xi_{1}\\ \xi_{2} \end{array}\right]=B\left[\begin{array}{l} \rho \\ I \end{array}\right]+\xi$$

where $B=\left[\begin{array}{cc} 1 & -k_{1}\\ 1 & -k_{2} \end{array}\right]$ and $\xi ={\left[\xi_{1},\xi_{2}\right]}^{T}$. The ionospheric delay can be expressed as follows:

(9) $$\left[\begin{array}{l} \hat{\rho }\\ \hat{I} \end{array}\right]={\left(B^{T}B\right)}^{-1}B^{T}L$$

The coefficients of the ionospheric delays are assumed as ***b***. Thus, the estimated ionospheric delays can be written as:

(10) $$\hat{I}=b^{T}L$$

However, the variations of the total electron content (TEC) could be assumed as a polynomial model during a short time (one hour) given an inactive ionosphere period, which can be concluded from the estimated errors of ionospheric delays (Figure 4). Therefore, this study takes a polynomial function to fit the ionospheric delays in short time (five epochs). Thus:

(11) $${\hat{I}}_{\left(t\right)}=\theta_{0}+\theta_{1}t+\theta_{2}t^{2}+e_{m}^{'}$$

where θ is the polynomial coefficients; *t* is the epoch point, and $e_{m}^{'}$ is the model fitting residual. The ionospheric delay is assumed to be fitted by five epochs. Then:

(12) I=Gθ+dI

where $I={\left[\begin{array}{ccccc} {\hat{I}}_{\left(t-5\right)} & {\hat{I}}_{\left(t-4\right)} & {\hat{I}}_{\left(t-3\right)} & {\hat{I}}_{\left(t-2\right)} & {\hat{I}}_{\left(t-1\right)} \end{array}\right]}^{T}$, $\theta ={\left[\begin{array}{ccc} \theta_{0} & \theta_{1} & \theta_{2} \end{array}\right]}^{T}$, $dI={\left[\begin{array}{ccc} {\left(e_{m}^{'}\right)}_{1} & \cdots & {\left(e_{m}^{'}\right)}_{5} \end{array}\right]}^{T}$ and $G={\left[\begin{array}{ccccc} 1 & 1 & 1 & 1 & 1\\ -5 & -4 & -3 & -2 & -1\\ 25 & 16 & 9 & 4 & 1 \end{array}\right]}^{T}$

The coefficients of polynomial function are computed as follows:

(13) $$\hat{\theta }={\left(G^{T}G\right)}^{-1}G^{T}I$$

Then, the current epoch is set as *t* = 0. Therefore, the prediction of ionospheric delay is:

(14) $${\bar{I}}_{\left(t\right)}={\hat{\theta }}_{0}=g^{T}I$$

where $g={\left[\begin{array}{ccccc} g_{1} & g_{2} & g_{3} & g_{4} & g_{5} \end{array}\right]}^{T}$ represents the coefficients of five ionospheric delays. By inserting Equations (7) and (14) into Equation (6), we obtain:

(15) $$\begin{array}{l} \sigma_{\Delta \tilde{I}}^{2}=\mathrm{var}[e_{m}]+\mathrm{var}[d{\hat{\theta }}_{0}]+\mathrm{var}[d{\hat{I}}_{\left(t-1\right)}]-2\mathrm{cov}[d{\hat{\theta }}_{0},d{\hat{I}}_{\left(t-1\right)}]\\ =\sigma_{m}^{2}+(g^{T}g)(b^{T}b)\sigma_{L}^{2}+b^{T}b\sigma_{L}^{2}-2\mathrm{cov}[g_{5}d{\hat{I}}_{\left(t-1\right)},d{\hat{I}}_{\left(t-1\right)}]\\ =\sigma_{m}^{2}+[(g^{T}g)+1-2g_{5}](b^{T}b)\sigma_{L}^{2} \end{array}$$

(16) $$\begin{array}{l} c=\mathrm{cov}[\Delta \xi \Delta \tilde{I}]=\mathrm{cov}[-\xi_{\left(t-1\right)}d{\hat{\theta }}_{0}-d{\hat{I}}_{\left(t-1\right)}]\\ =\mathrm{cov}[-\xi_{\left(t-1\right)}(g_{5}-1)b^{T}\xi_{\left(t-1\right)}]=(1-g_{5})\sigma_{L}^{2}b \end{array}$$

In Equation (6), ***Q*** can be simplified as:

(17) $$Q=\sigma_{m}^{2}kk^{T}+\bar{Q}$$

where $\bar{Q}=\left\{(b^{T}b)[(g^{T}g)+1-2g_{5}]kk^{T}+E_{2}-(1-g_{5})kb^{T}-(1-g_{5})bk^{T}\right\}\sigma_{L}^{2}$ and $E_{2}$ is an identity matrix with two dimensions. Based on the matrix inversion lemma, ***Q*** can be expressed as:

(18) $$Q^{-1}={\bar{Q}}^{-1}-\frac{\sigma_{m}^{2}}{1+\sigma_{m}^{2}k^{T}{\bar{Q}}^{-1}k}{\bar{Q}}^{-1}kk^{T}{\bar{Q}}^{-1}={\bar{Q}}^{-1}-\theta_{m}M$$

where $M={\bar{Q}}^{-1}kk^{T}{\bar{Q}}^{-1}$ , and $\theta_{m}=\frac{\sigma_{m}^{2}}{1+\sigma_{m}^{2}k^{T}{\bar{Q}}^{-1}k}$.

The model error from Equations (15) to (18) is:

(19) $$\sigma_{m}^{2}(t)=(1-\mu )\sigma_{m}^{2}(t-2)+\mu ({\left[{\hat{I}}_{\left(t-1\right)}-{\bar{I}}_{\left(t-1\right)}\right]}^{2}-\sigma_{\bar{I}}^{2}(t-1))$$

whereμ is the impact factor of the previous epoch, and $\sigma_{\bar{I}}^{2}=(g^{T}g)(b^{T}b)\sigma_{L}^{2}$.

Therefore, in Equation (5), the solution of $A^{T}Q^{-1}A$ can be expressed as:

(20) $$A^{T}Q^{-1}A=A^{T}{\bar{Q}}^{-1}A-\theta_{m}A^{T}MA$$

According to the above equations, the cycle-slip values on *L*_2_ can be calculated accurately by inserting Equation (20) into Equation (5). Moreover, inserting the fractional parts on *L*_2_ adjusted to integer numbers into Equation (3), can solve the cycle slips on *L*_1_ observations. In the improved cycle-slip detection and repair algorithm, the estimated ionospheric delay in Equation (10) contains the constant biases, namely, integer ambiguity and hardware delay in adjacent epochs, which do not affect the cycle-slip detection at all.

In this study, an improved algorithm of double-frequencies’ cycle-slip detection and repair is proposed. The new algorithm considers the drawbacks of the inaccurate traditional Turboedit (see Table 4) to refine the data preprocessing based on a predicted ionospheric delay. A polynomial prediction model is used to acquire the ionospheric delays of the current epoch, which takes the correlation of adjacent epochs into consideration. Moreover, the cycle slips are detected and repaired epoch-wise to increase data availability. In general, compared with the traditional Turboedit, the advantages of the improved algorithm can be summarized as follows: (1) the algorithm, which is based on the predicted ionospheric delay information rather than a geometry-free (GF) combination, solves the problem of special cycle-slip combinations in two frequencies (such as 1:1 and 9:7 for *L*_1_:*L*_2_ in GPS observations); (2) the improved algorithm is more accurate than MW and GF combinations in Turboedit, which has to fit the GF and ignores the correlation of the ionospheric delays; (3) in the estimation of ionospheric delays, the pseudo-range observations, which have an impact on the detection and repair of the small cycle-slips as the larger noise than phase observations, are disregarded; and (4) the variations in the ionospheric delay estimation caused by constant biases in Equation (14) are zero when $\sum_{i=1}^{5}g_{i}=1$ ($\delta \Delta I_{t,t-1}=\delta {\bar{I}}_{\left(t\right)}-\delta {\bar{I}}_{\left(t-1\right)}=g^{T}\delta I-\delta {\hat{I}}_{\left(t-1\right)}=(\sum_{i=1}^{5}g_{i}-1)b^{T}\delta N=0$).

**Figure 1:**
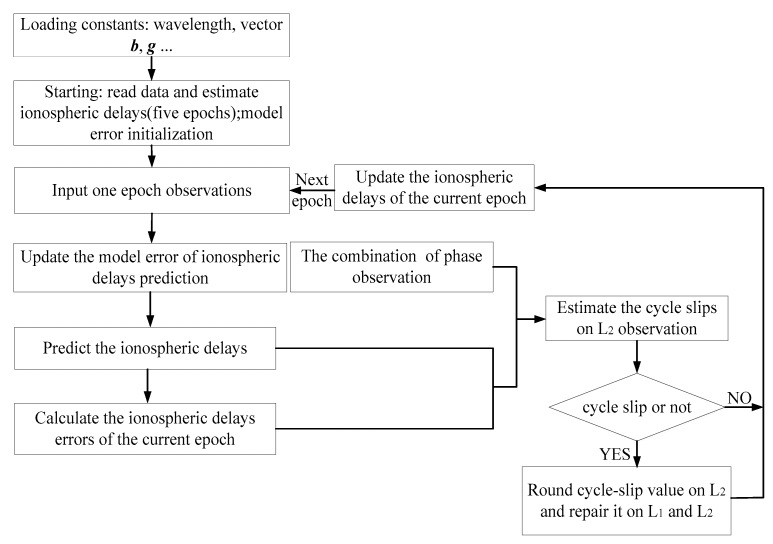
Flowchart of the improved cycle-slip detection and repair algorithm.

To illustrate the improved cycle-slip detection and repair algorithm, Figure 1 shows the flow chart of the above procedure. Moreover, the data from iGMAS and MGEX tracking networks are selected to verify the reliability and availability of the improved algorithm. The fractional part of the estimated *L*_2_ based on the improved algorithm is extracted by no cycle-slip observations of iGMAS (WHU1 and LHA1) and MGEX (LHAZ). Four days (DOY 183–186, 2017) of observations with a 30 s sampling interval were chosen to conduct the cycle-slip detection and repair experiments and then to fully explain the accuracy of the improved algorithm. Due to the fact that the experimental datasets were extremely large, the G01 and C01, C06, C14, and C32 were selected as the representative of GPS, and GEO, IGSO, MEO, and BDS-3 in BeiDou to analyze the results, respectively. Table 2 summarizes the maximum, average, and standard deviation (STD) of the fractional parts of the estimated cycle slips and the properness after rounding on G01. Similarly, results of BeiDou satellites (C06 missed on DOY 184) are listed in Table 3, in which only LHA1 is listed to conduct the corresponding analysis.

In Table 2, the average and STD of the fractional parts of the estimated *L*_2_ are less than 0.2 cycles, whereas some of the maximum values are beyond 0.5 cycles, especially for WHU1. Moreover, based on the improved algorithm, a few wrong cycle slips were found in this study. However, the properness is beyond 99% for all experiments, in which the incorrect estimation for iGMAS is slightly higher than for MGEX observations. It can be explained that the larger noise of iGMAS observations than for MGEX causes the inaccurate estimation of ionospheric delay from the analysis of Table 1.

**Figure 2:**
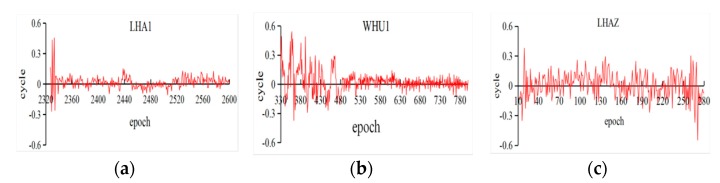
Time series of the fractional parts of *L*_2_ observations for G01 on DOY 183 ((**a**) LHA1; (**b**) WHU1; and (**c**) LHAZ).

**Figure 3:**
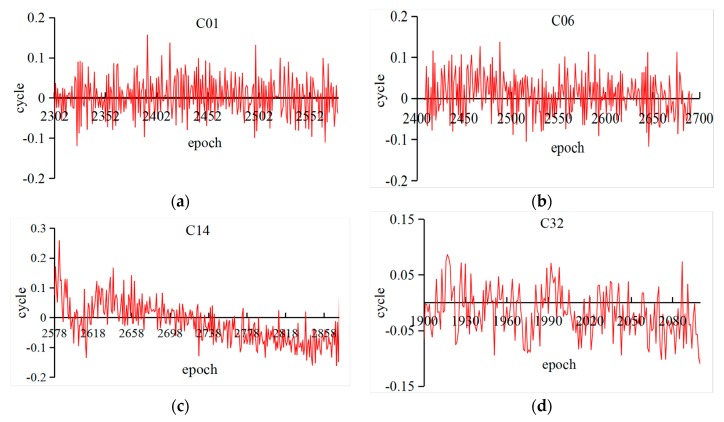
Time series of the fractional parts of B2 (C01, C06, C14) and B3 (C32) observations on DOY 183 based on LHA1 ((**a**) C01 (BDS_GEO); (**b**) C06 (BDS_IGSO); (**c**) C14 (BDS_MEO); and (**d**) C32 (BDS-3)).

In Table 3, the properness of four days for different BeiDou satellites on LHA1 reached upwards of 100%. Meanwhile, the maximum values are smaller than for G01 in Table 2, which can ensure the properness of rounding the fractional parts. However, it should be noted that the results of C32 seem to be better than for BDS-2. According to the experiments, it proved that the improved algorithm is reliable for BeiDou observations, especially for BDS-3 experimental satellites. Furthermore, the fractional parts of estimated cycle slips in all epochs are demonstrated in Figure 2 and Figure 3 to provide the details of the improved algorithm.

In Figure 2, the fractional parts of the G01 (DOY 183) at three stations are drawn; it could be found that the fractional parts of iGMAS observations are convergent as the epoch increases. Since the improved algorithm is epoch-wise processing, the results are influenced by the noise of observations at the beginning. In addition, the MGEX observations are almost less than 0.3 cycles. Similarly, Figure 3 shows the results of three types BeiDou satellites and BDS-3 experimental satellites, which are based on LHA1 observations on DOY 183. Simultaneously, the corresponding fractional parts are less than 0.2 cycles. However, the result was slightly worse for MEO than for IGSO and GEO satellites and its trend could be caused by the BeiDou satellite-induced biases [31]. The accuracy of the improved algorithm proposed in this study can meet the requirements of orbit determination by testing iGMAS and MGEX observations (without cycle slips). The properness of rounding reached 100%, except for a few errors.

**Figure 4:**
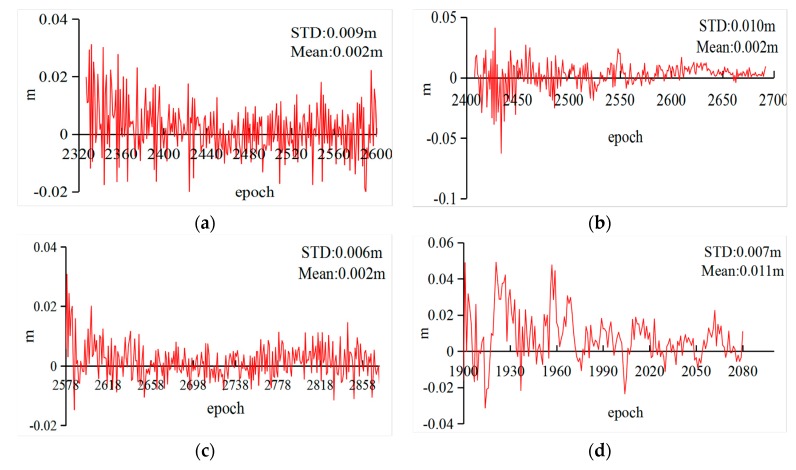
The differences between the estimated and predicted ionospheric delays ((**a**) G01; (**b**) C06; (**c**) C14; and (**d**) C32).

Then, a group of cycle slips was inserted into the observations to test the reliability of the improved algorithm. Due to the fact that the Turboedit could not solve the special combinations on different frequencies, such as 1:1 or 9:7 for cycle slips on *L*_1_ and *L*_2_ of GPS, the experiment selected six types of combinations ((0,1), (1,0), (1,1), (9,7), (100,1), and (790,563)) to analyze the improved algorithm [32]. The observations from the last experiment were inserted cycle slips, and the repaired results are based on rounding the float estimates. Table 4 only summarizes the results of G01, C01, C06, C14, and C32 based on LHA1 on DOY 183. In Table 4, the cycle slips in GPS or BeiDou observations, correspondingly, were completely corrected based on the improved algorithm. However, the estimated cycle slips at epoch 2600 were (0.525,0.698), which is difficult to fix as the real values in the ambiguity resolution. In this study, from the results of all experiments, this phenomenon need not be taken into consideration as the very few numbers did not impact the corresponding conclusions. Moreover, to further analyze the improved algorithm, the results of Turboedit were also listed in Table 4. The experimental results show that the ability of the improved algorithm to detect and repair cycle slips outperforms the Turboedit approach, especially for repairing values. Furthermore, the differences between the estimated and the predicted ionospheric delays are also calculated to test the accuracy of the predicted polynomial model. Figure 4 displays the ionospheric delay residuals for G01, C06, C14, and C32 (LHA1) calculated from the same data as Figure 2. In Figure 4, the errors of the predicted ionospheric delays are below 0.02 m as time increases, which can meet the requirements of the cycle-slip detection and repair.

The simulation experiments verified the accuracy of the improved cycle-slip detection and repair algorithm proposed in this study. The orbit determination should be further tested to demonstrate the reliability of the proposed algorithm. However, given the data quality and limited data sources, the observations from multiple tracking networks must be fully applied to improve the accuracy of the parameter estimation. In the following section, two methods were used to effectively utilize the observations from iGMAS and MGEX tracking networks.

## 3. Two Methods for BDS-3 Orbit Determination

Concerning observations two factors must be considered in BDS-3 orbit determination: (1) Due to the restriction of the number and distribution of BDS-3 tracking stations, it is insufficient for BDS-3 observations required in BDS-3 orbit determination. As visible in Figure 7, the number of BDS-3 tracking stations increased from 9 to 17 at the end of the experiment period. According to the station distribution theory [33], this number of stations was far from meeting the precise orbit determination requirements; (2) The observation types from different tracking networks were different from each other, especially for BeiDou. Based on the analysis of the observations from iGMAS and MGEX, BDS-2 (mainly B1 and B2) could be received by the MGEX and iGMAS, while BDS-3 was only acquired by iGMAS with B1 and B3. Therefore, the orbit determination suffered from the inconsistency between two tracking networks. However, in the study of multi-GNSS orbit determination [19], the full use of various observations improved the strength of the parameter estimation and indirectly enhanced the accuracy of the orbit parameters. Therefore, two methods, namely, NES and step-by-step (SS), for improving the accuracy of BDS-3 orbit estimations using the combination of different networks, were proposed.

### 3.1. BDS-3 Orbit Determination Based on NES

The parameters related to the BDS-3 experimental satellites orbits could be improved indirectly through the MGEX observations, which accurately obtains the relevant parameters (station coordinates, tropospheres, and clock offsets). This study improved the data availability based on the NES of two networks in BDS-3 orbit determination.

The normal equation stacking method has been well discussed [27,34]. In this study, based on the orbit determination, some more details about NES were given as follows:

The normal equations of BDS-3 orbit determination based on iGMAS observations can be assumed according to [27]:

(21) $$\left[\begin{array}{cc} N_{11} & N_{12}\\ N_{21} & N_{22} \end{array}\right]\left[\begin{array}{l} X\\ Y \end{array}\right]=\left[\begin{array}{l} W_{1}\\ W_{2} \end{array}\right]$$

where *X* consists of the parameters of troposphere, station coordinates, and stations clocks; *Y* denotes the parameters related to the BDS-3, such as orbit parameters, satellites clock offsets; and *N* is the coefficient matrix corresponding to the normal equation.

Similarly, the normal equations of the combined GPS and BDS-2 orbit determination based on MGEX and iGMAS are set up as in [27]:

(22) $$\left[\begin{array}{cc} N_{11}^{'} & N_{12}^{'}\\ N_{21}^{'} & N_{22}^{'} \end{array}\right]\left[\begin{array}{l} X\\ Y^{'} \end{array}\right]=\left[\begin{array}{l} W_{1}^{'}\\ W_{2}^{'} \end{array}\right]$$

where *X* represents the same parameters, such as iGMAS station coordinates, troposphere, and stations clocks, as in Equation (21). *Y′* is the station-related MGEX as mentioned above (MGEX station coordinates, troposphere, and stations clocks), and GPS and BDS-2 satellite orbit parameters based on iGMAS and MGEX networks.

Thus, the estimation of *X* is obtained using Equations (21) and (22) [27]:

(23) $$\left(N_{11}-N_{12}N_{22}^{-1}N_{21}\right)X=W_{1}-N_{12}N_{22}^{-1}W_{2}$$

(24) $$[N_{11}^{'}-N_{12}^{'}{\left(N_{22}^{'}\right)}^{-1}N_{21}^{'}]X=W_{1}^{'}-N_{12}^{'}{\left(N_{22}^{'}\right)}^{-1}W_{2}^{'}$$

Combining Equations (23) and (24), then, assuming $Q=N_{11}+N_{11}^{'}$, $M=N_{12}N_{22}^{-1}N_{21}+N_{12}^{'}{\left(N_{22}^{'}\right)}^{-1}N_{21}^{'}$, $W=W_{1}^{'}+W_{1}$, and $K=N_{12}N_{22}^{-1}W_{2}+N_{12}^{'}{\left(N_{22}^{'}\right)}^{-1}W_{2}^{'}$:

(25) [Q−M]X=W−K

In Equation (25), *X* is based on the inversion of [*Q–M*]. This study proposes a simple method to improve computational efficiency and avoid program overflow caused by large matrix dimensions, as follows:

The symmetric matrix *Q* is decomposed by the Cholesky algorithm as $Q=R^{T}R$. *R* is a low triangular matrix:

(26) $$Q-M=R^{T}(R^{-T}(Q-M)R^{-1})R=R^{T}[E-\bar{M}]R$$

where $\bar{M}=R^{-T}MR^{-1}$. The eigenvalue decomposition of $\bar{M}$ is used to obtain:

(27) $$\bar{M}=V^{T}DV$$

In Equation (27), *V* is the orthogonal matrix after decomposition, and $D=diag(\begin{array}{cccc} d_{1} & d_{2} & \cdots & d_{n} \end{array})$. Thus:

(28) $$Q-M=R^{T}V^{T}(E-D)VR=U^{T}(E-D)U$$

where U=RV. Set *X* as *n* dimensions. *E* is an identity matrix with *n × n* elements. Then, in Equation (28):

(29) $${\left[Q-M\right]}^{-1}=U^{-1}\left[\begin{array}{ccccc} \frac{1}{1-d_{1}} & & & & \\ & \frac{1}{1-d_{2}} & & & \\ & & \ddots & & \\ & & & \frac{1}{1-d_{n-1}} & \\ & & & & \frac{1}{1-d_{n}} \end{array}\right]U^{-T}$$

In Equation (29), set:

(30) $$U^{-1}=\left[\begin{array}{cccc} {\bar{u}}_{11} & {\bar{u}}_{12} & \cdots & {\bar{u}}_{1n}\\ {\bar{u}}_{21} & {\bar{u}}_{22} & \cdots & {\bar{u}}_{2n}\\ \vdots & \vdots & \ddots & \vdots \\ {\bar{u}}_{n1} & {\bar{u}}_{n2} & \cdots & {\bar{u}}_{nn} \end{array}\right]$$

Thus, *X_i_* can be further simplified, if:

(31) $$u_{m}={\left[\begin{array}{ccccc} \frac{{\bar{u}}_{i1}}{1-d_{1}} & \frac{{\bar{u}}_{i2}}{1-d_{2}} & \cdots & \frac{{\bar{u}}_{ii}}{1-d_{i}} & \frac{{\bar{u}}_{in}}{1-d_{n}} \end{array}\right]}^{T}$$

then:

(32) $${\hat{X}}_{i}=u_{m}^{T}U^{-T}(W-K)$$

The relevant parameters about the BDS-3 orbits can be solved by substituting Equation (32) into (21). Thus:

(33) $$Y=N_{22}^{-1}(W_{2}-N_{21}\hat{X})$$

**Figure 5:**
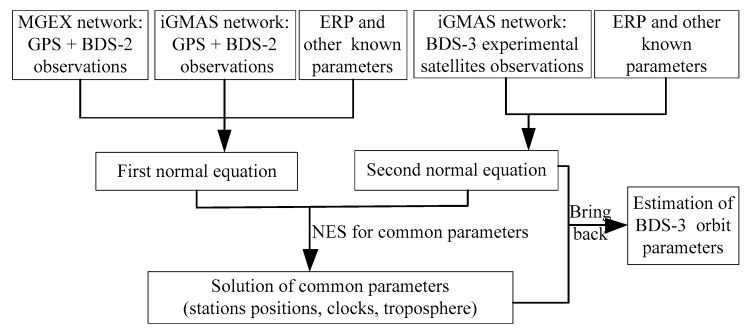
Flowchart of BDS-3 orbit determination based on NES.

The BDS-3 orbit parameters can be determined by the same methods as mentioned above, and three-day arcs can be obtained by the corresponding state transformation matrix. Figure 5 illustrates the flowchart of NES to specifically describe the method of BDS-3 orbit determination.

Since the parameters of the ISBs and IFBs between GPS and BDS-2 in the combined orbit determination are highly correlated, the biases are introduced as one type of estimated parameter to avoid separating them from each other. However, as the characteristics of bias parameters for the BDS-3 system are not taken into consideration in the current research, the biases between the BDS-3 and GPS (BDS-2) are difficult to define in the combined orbit determination. Therefore, as shown in Figure 5, additional transformation parameters (spatial seven-parameter transformation) are set to adjust between two normal equation systems.

### 3.2. BDS-3 Orbit Determination Based on SS

**Figure 6:**
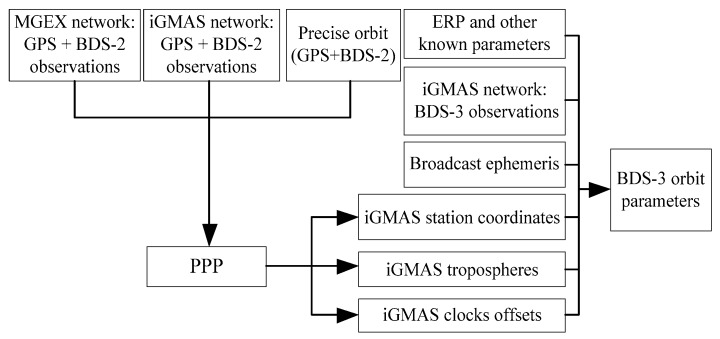
Flowchart of BDS-3 orbit determination based on SS.

The BDS-3 experimental satellite orbit determination based on NES is an effective method to avoid the observation inconsistency of the different tracking networks caused by different tracking signals. Similarly, the fixed parameters, such as station coordinates, tropospheric delays, and clock offsets of the iGMAS stations, can be introduced into the BDS-3 orbit determination by precise point positioning (PPP). Therefore, the SS method is also adopted in this study. Figure 6 depicts the flowchart of BDS-3 experimental satellite orbit determination based on iGMAS and MGEX networks by SS.

Two methods for improving the accuracy of BDS-3 experimental satellites orbit estimations were provided by using different tracking networks. The NES contains more information on observations with the same data sources as the SS, which is more precise (in Section 4, as the perfect modeling of the stochastics, the orbit results estimated by the NES are more accurate and precise than the SS) in the parameter estimation. Based on the improved cycle-slip detection and repair algorithm and the two methods of orbit determination, data quality, and availability considerably improved given the limited number of observations. The precise orbit determination and the corresponding accuracy analysis are discussed in the next section.

## 4. BDS-3 Orbit Determination and Its Accuracy Analysis

**Figure 7:**
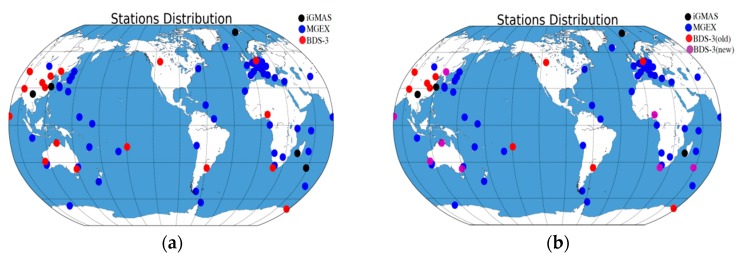
Stations distribution of BDS-3 experimental satellites orbit determination (black: iGMAS stations without BDS-3 observations; red: iGMAS stations with BDS-3 observations; blue: MGEX stations; and magenta: increased iGMAS stations with BDS-3 observations). (**a**) Stations distribution at the beginning, (**b**) Stations distribution at the end.

In this study, the BDS-3 experimental satellite orbit determination was conducted from DOY 154, 2016 to DOY 149, 2017 with iGMAS and MGEX observations. The distribution of tracking stations is presented in Figure 7a, where the types of stations are represented by different colors. A total of 16 iGMAS (nine of them contain BDS-3 observations) and 54 MGEX stations were used at the beginning of the orbit determination. However, numbers of stations containing BDS-3 observations increased to 17 at the end of the experiment given the equipment update and network extension of the iGMAS. Figure 7b shows the distribution of stations, where the BDS-3 tracking stations are presented with magenta. The parameter configurations and related models of orbit determination refer to [15], in which the main parameters are listed in Table 5. Moreover, the model used in used GPS orbit determination, e.g., for solar radiation pressure, were considered here as a reference.

**Figure 8:**
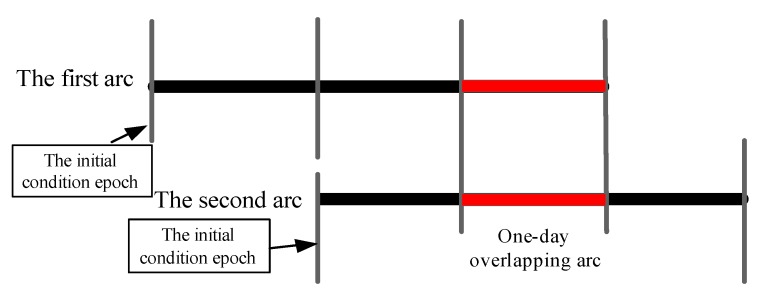
Sketch of the orbit overlapping arcs.

This study assessed the BDS-3 experimental satellite orbit based on the following experiments to adequately analyze its accuracy: (1) the 3D RMS of one-day overlapping arc errors based on two adjacent orbit determination processes, the scheme of which is displayed in Figure 8; and (2) BDS-3 is equipped with laser retroreflector array, which can be used to check the orbit accuracy by SLR residuals.

**Figure 9:**
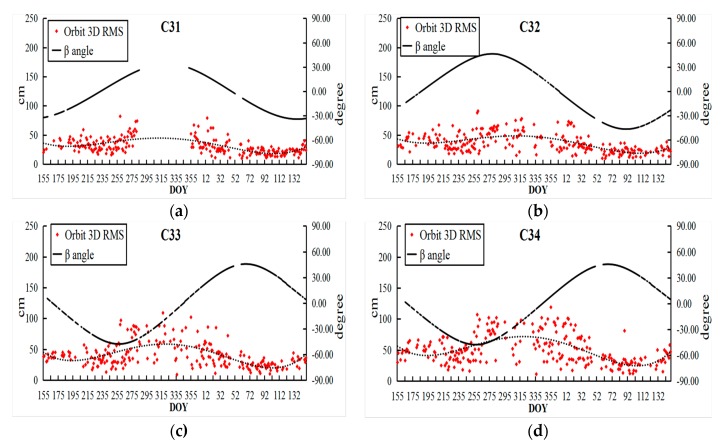
The 3D RMS of BDS-3 one-day overlapping arcs errors based on NES ((**a**) C31; (**b**) C32; (**c**) C33; and (**d**) C34).

**Figure 10:**
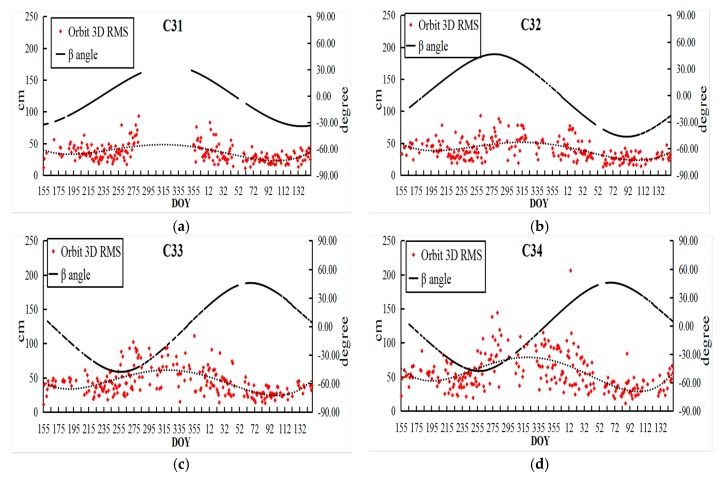
The 3D RMS of BDS-3 one-day overlapping arc errors based on SS ((**a**) C31; (**b**) C32; (**c**) C33; and (**d**) C34).

The overlapping arcs accuracy of two methods for BDS-3 (C31, C32, C33, and C34) orbit determinations are illustrated in Figure 9 and Figure 10 for the period DOY 155, 2016 until DOY 149 in 2017. The variations of the orbit accuracy were fitted by a trend line (dashed) in the results. In addition, the angles between orbital plane and Sun called β were also plotted in corresponding figures.

In Figure 9 and Figure 10, the accuracy of BDS-3 was worse at the end of 2016, while it became better in 2017 due to the update of the iGMAS tracking network. Moreover, it was found that the β angles were highly-correlated with the accuracy of the BDS-3, especially for IGSO (C31 and C32). Because the solar radiation pressure model of GPS was taken into the orbit determination, this is not suitable for the new-generation BeiDou satellites and should be refined in later studies.

**Figure 11:**
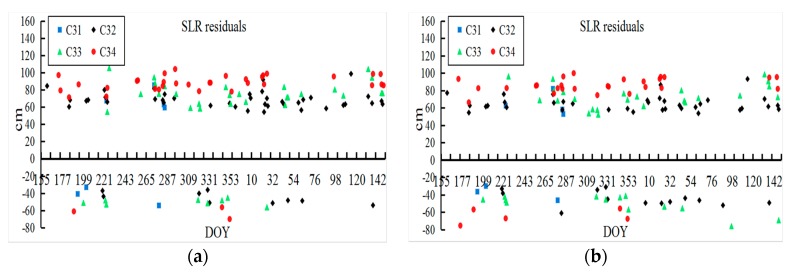
The SLR residuals of BDS-3 orbits based on NES and SS methods. (**a**) NES method, (**b**) SS method.

In addition, the SLR residuals of the two methods are shown in Figure 11. The corresponding mean and RMS values of SLR residuals are presented in Table 6 based on the one-year BDS-3 orbit determination to analyze the orbit accuracy by the NES and SS methods. Since the SLR data points of BDS-3 were insufficient, the trend of the SLR residuals could not be acquired accurately.

In the experiments, the 3D RMS of one-day overlapping arcs of BDS-3 (C31, C32, C33, and C34) based on the NES and SS are 31.0, 36.0, 40.3, and 50.1 cm and 34.6, 39.4, 43.4, and 55.5 cm, respectively. The RMS of SLR residuals of the NES and SS are 55.1, 49.6, 61.5, and 70.9 cm and 60.5, 53.6, 65.8, and 73.9 cm, respectively. In addition, the mean values of SLR residuals for the two methods are approximately equal, which could be caused by the different laser retroreflector arrays between BDS-2 and BDS-3. The experimental results showed that the NES is slightly better than the SS in BDS-3 orbit determination. Furthermore, the reason why the NES offers more accurate BDS-3 orbits can be explained as follows: (1) Due to the restriction of the number and distribution of BDS-3 tracking stations, the correlations between the BDS-3 orbit parameters and iGMAS stations related parameters are clearer. However, the SS method ignores the stochastic information of iGMAS parameters in the final parameter determination step; (2) The biases between different types satellites cannot be eliminated in SS, while the NES method takes the spatial seven-parameter transformation to reduce the difference of two normal equation. Moreover, the results in Table 6 show that the mean and RMS values of SLR residuals are larger than the initial assessment results conducted in [13]. It is suggested that a system error caused by the different SLR-related parameters (SLR retroreflectors) is included in the final results.

Then, one-month of observations (DOY 228–259, 2016) were selected to conduct the BDS-3 orbit determination with four schemes and further illustrate the reliability and advantages of the proposed methods. This study calculated the orbit accuracy of BDS-3 in four schemes according to orbit overlapping arc errors. The specific schemes are as follows:

Scheme 1: Based on the BDS-3 orbit determination using iGMAS observations, the traditional Turboedit was used, and the BDS-3 orbits were determined by one step (compared with SS, one step is widely used by GNSS data analysis centers [35], which estimates all parameters in a single-step solution).
Scheme 2: Based on iGMAS observations, the improved cycle-slip detection and repair algorithm was used, and the BDS-3 orbits were acquired through one step.
Scheme 3: Based on iGMAS and MGEX observations, the improved cycle-slip detection and repair algorithm was used to determine the BDS-3 orbits through SS.
Scheme 4: Based on iGMAS and MGEX observations, NES based on the improved cycle-slip detection and repair proposed in this study was used to determine the BDS-3 orbits.

The SLR residuals of the BDS-3 orbit cannot be accurately determined because the limited SLR data within the experimental period. Therefore, this study only counted the discrepancy of overlapping arcs as illustrated in Figure 8. Similarly, Table 7 lists the corresponding orbit 3D RMS and improvement (compared with Scheme 1), in which Scheme 4 was considered as the optimal strategy. Moreover, the accuracy of the BDS-3 orbits obtained by Scheme 4 improved by 34.07%, 41.05%, 72.29%, and 74.33% for C31, C32, C33, and C34, respectively. However, the improvement of orbit accuracy using Scheme 2 was less distinct than Scheme 3 and Scheme 4, which indicated that the contribution of the improved cycle-slip detection and repair algorithm to the parameter estimation was less than the combination of different observation networks, given the fact that the combination offers a more reasonable global network structure. The orbit accuracy obtained using Scheme 3 was slightly worse than that of Scheme 4; this result is consistent with the theory discussed earlier. Furthermore, to analyze the reliability of the improved cycle-slip detection and repair algorithm, the data availability was taken into consideration based on the residuals of every epoch in Scheme 2. In Table 8, the threshold was set as 4σ, where σ is the mean square error of observation residuals, to eliminate the observations with poor quality. From Table 8, the improved algorithm enhanced the data availability from 12.90% to 20.46% compared with the traditional Turboedit approach.

## 5. Conclusions and Prospects

In this study, the BDS-3 experimental satellite’s precise orbit determination and the corresponding analyses of the results were conducted based on the iGMAS and MGEX networks. In the data processing, an improved cycle-slip detection and repair algorithm was proposed to overcome the disadvantages of the traditional Turboedit in the iGMAS data. From the analysis of observations, it is suggested that the new algorithm is reliable and efficient in improving the data availability of iGMAS observations and accuracy of the parameters related to BDS-3, which considers the correlation of ionospheric delays between adjacent epochs based on the polynomial prediction. Moreover, the improved algorithm preprocesses observations epoch-wise and can optimize the observations in real-time.

The insufficient BDS-3 observations caused by the tracking stations were the major factor for limiting its orbit accuracy. In this study, the MGEX observations were used to enhance the estimation of the iGMAS station-related parameters, which indirectly improved the accuracy of the BDS-3 orbit parameters. However, the observation types from the iGMAS for BDS-3 were B1 and B3, while MGEX mainly tracked B1 and B2 for BDS-2. Therefore, this study designed two BDS-3 orbit determination methods, namely, NES and SS, to achieve the combination of different observations. In NES, the common parameters were obtained through the Gaussian elimination method based on the normal equation from the BDS-3 orbit determination with iGMAS, and that of GPS and BDS-2 orbit determination with iGMAS and MGEX. However, SS was utilized to fix the iGMAS station-related parameters by the PPP network solution with GPS and BDS-2 observations. Then, the fixed parameters were assumed as the known parts in the BDS-3 orbit determination. Moreover, a matrix decomposition method was proposed in this study to improve the efficiency of the parameter estimation in NES.

One-year BDS-3 (C31, C32, C33, and C34) orbit determination based on the MGEX and iGMAS observations were conducted, and the corresponding orbit accuracy of four satellites were analyzed. The accuracy of BDS-3 was calculated from the discrepancy of the overlapping arcs and SLR residuals. Results showed that BDS-3 orbit accuracy obtained through the NES and SS were 31.0, 36.0, 40.3, and 50.1 cm and 34.6, 39.4, 43.4, and 55.5 cm, respectively. Meanwhile, the RMSs of the SLR residuals were 55.1, 49.6, 61.5, and 70.9 cm and 60.5, 53.6, 65.8, and 73.9 cm, respectively. The accuracy of the BDS-3 orbit was gradually improved by fitting the trend of overlapping arcs, which was mainly related to the increased number of BDS-3 stations in the iGMAS network. In addition, it was found that the orbit accuracy was highly-correlated with the β angle, especially for IGSO (C31 and C32), during the experimental period.

Four schemes of BDS-3 orbit determination were designed by one-month observations to fully illustrate the reliability and advantages of the improved strategies proposed in this study. The orbit accuracy and improvement of each scheme were calculated. Results showed that the NES based on the improved cycle-slip detection and repair was optimal for BDS-3 orbit determination compared with other schemes. The accuracy of the BDS-3 orbit improved by 34.07%, 41.05%, 72.29%, and 74.33%, for C31, C32, C33, and C34, correspondingly.

However, the limited tracking observations and unknown satellite parameters resulted in low accuracy in BDS-3. Therefore, the ISL and low Earth orbit satellites will be considered in the orbit determination to further improve the accuracy of BDS-3 orbit in the follow-up research tasks. Moreover, the application of precise BDS-3 orbits based on the new signals will be discussed as given in [36,37].

## Figures and Tables

**Table 1:** Comparison of data quality between iGMAS and MGEX stations.

Stations		GPS				BDS-2		BDS-3	
		Effective Rate	MP1 (m)	MP2 (m)	CSR	Effective Rate	CSR	Effective Rate	CSR
MGEX	JFNG	88.80%	0.348	0.424	681.20	99.66%	118.01	78.24%	843.11
iGMAS	WHU1	81.60%	0.365	0.472	133.85	85.47%	1160.23	80.22%	942.25
MGEX	LHAZ	90.61%	0.418	0.344	239.40	95.25%	77.94	94.51%	334.23
iGMAS	LHA1	99.56%	0.473	0.370	128.06	84.99%	4393.38	99.61%	439.50

**Table 2:** The fractional part of estimated *L*_2_ observations on G01 (cycle).

Stations	DOY	Maximum	Average	STD	Properness
LHA1	183	0.469	0.013	0.114	100%
	184	0.242	0.006	0.091	100%
	185	0.366	0.006	0.113	100%
	186	0.422	0.029	0.141	100%
LHAZ	183	0.546	0.003	0.125	99.92%
	184	0.201	0.045	0.064	100%
	185	0.307	0.089	0.086	100%
	186	0.373	0.033	0.072	100%
WHU1	183	0.577	0.013	0.133	99.92%
	184	0.322	0.051	0.092	100%
	185	0.572	0.005	0.137	99.68%
	186	0.505	0.001	0.133	99.84%

**Table 3:** The fractional parts of the estimated B2 observations for C01, C06, C14 (BDS-2), and B3 for C32 (BDS-3) on LHA1 (cycle).

DOY	Satellites	Maximum	Average	STD	Properness
183	C01	0.178	0.003	0.125	100%
	C06	0.137	0.001	0.051	100%
	C14	0.259	0.013	0.071	100%
	C32	0.129	0.014	0.094	100%
184	C01	0.201	0.045	0.064	100%
	C06	-	-	-	-
	C14	0.259	0.019	0.068	100%
	C32	0.183	0.056	0.106	100%
185	C01	0.089	0.307	0.087	100%
	C06	0.212	0.023	0.058	100%
	C14	0.239	0.083	0.066	100%
	C32	0.166	0.081	0.138	100%
186	C01	0.373	0.033	0.072	100%
	C06	0.196	0.035	0.045	100%
	C14	0.386	0.051	0.125	100%
	C32	0.148	0.033	0.137	100%

**Table 4:** Results of GPS (G01), three types of BDS-2 (C01, C06, C14), and the BDS-3 experimental satellite (C32) based on the improved algorithm and Turboedit for DOY 183 in LHA1 (cycle).

Satellite	Epoch	Cycle Slips (L_1_,L_2_), (B1,B2) or (B1,B3)	Improved Algorithm				Turboedit			
			Estimated	ΔN_1_	ΔN_2_ (ΔN_3_)	True or False	Estimated	ΔN_1_	ΔN_2_ (ΔN_3_)	True or False
G01	2400	(0,1)	(−0.037,1.113)	0	1	T	(0.486,0.302)	0	0	F
	2500	(1,0)	(0.908,0.031)	1	0	T	(0.442,0.334)	0	0	F
	2600	(1,1)	(0.525,0.698)	1	1	T	(0.301,0.755)	0	1	F
	2600	(9,7)	(8.977,6.969)	9	7	T	(9.986,6.421)	10	6	F
	2500	(790,563)	(789.972,563.108)	790	563	T	(763.865,588.601)	764	589	F
	2400	(100,1)	(99.989,1.012)	100	1	T	(99.856,0.581)	100	1	T
C01	100	(0,1)	(0.042,1.063)	0	1	T	(0.014, 0.882)	0	1	T
	150	(1,0)	(0.975,−0.006)	1	0	T	(0.745,1.639)	1	2	F
	210	(1,1)	(1.195,1.161)	1	1	T	(1.802,1.930)	2	2	F
	150	(790,563)	(789.975,563.205)	790	563	T	(765.338,589.172)	765	589	F
	150	(100,1)	(99.743,0.934)	100	1	T	(99.663,1.015)	100	1	T
C06	2500	(0,1)	(0.083,1.071)	0	1	T	(0.293,0.985)	0	1	T
	2600	(1,0)	(1.029,0.017)	1	0	T	(1.633,0.441)	2	0	F
	2700	(1,1)	(1.047,0.975)	1	1	T	(1.112,0.994)	1	1	T
	2700	(790,563)	(790.221,562.909)	790	563	T	(788.920,568.202)	789	568	F
	2700	(100,1)	(100.023,0.805)	100	1	T	(101.043,0.189)	101	0	F
C14	2500	(0,1)	(0.029,1.045)	0	1	T	(0.254,0.935)	0	1	T
	2600	(1,0)	(0.941,0.019)	1	0	T	(1.338,0.014)	1	0	T
	2700	(1,1)	(0.995,1.024)	1	1	T	(1.733,0.696)	2	1	F
	2700	(790,563)	(789.891,562.990)	790	563	T	(762.284,589.445)	762	589	F
	2700	(100,1)	(99.987,0.902)	100	1	T	(98.472,0.809)	98	1	F
C32	1950	(0,1)	(0.031, 0.992)	0	1	T	(0.044,1.021)	0	1	T
	2000	(1,0)	(1.043,−0.012)	1	0	T	(0.994,0.852)	1	1	F
	2050	(1,1)	(1.113,1.093)	1	1	T	(1.442,0.843)	1	1	T
	2050	(790,563)	(789.998,563.014)	790	563	T	(764.745,566.233)	765	566	F
	2050	(100,1)	(99.940,0.982)	100	1	T	(99.493,0.984)	99	1	F

**Table 5:** Parameter configurations for BDS-3 experimental satellite orbit determination.

Parameter Name	Configuration
Observation	Undifferenced ionosphere-free code and phase combination B1 and B2 (BDS-2); B1 and B3 (BDS-3); L1 and L2 (GPS)
Elevation cut-off	5°
Weighing strategy	Elevation-dependent for the observation below 30° by 1/2sin (E)
Observation sample interval	30 s
Arcs length	three-days
Receiver ISB and IFB	Estimation: BDS-2 and GPSBDS-3: without consideration
Satellite phase center offset	BeiDou [15]; GPS: igs_08.atx
Tide models	International Earth Rotation and Reference Systems (IERS) 2010
Relativity	IERS 2010
Gravity model	EGM 08 12 × 12
Satellite phase center variation	BeiDou: without consideration; GPS: igs_08.atx
Satellite yaw models	BDS-3 experimental satellites: Nominal model; BDS-2 and GPS: reference to [9]
Solar radiation pressure models	ECOM
Ground antenna PCO and PCV	Not applied

**Table 6:** BDS-3 orbit accuracy of the two methods based on one-year observations (cm).

Satellites	3D RMS of One-Day Overlapping Arcs		SLR Residuals			
	NES	SS	NES		SS	
			Mean	RMS	Mean	RMS
C31	31.0	34.6	20.4	55.1	21.0	60.5
C32	36.0	39.4	35.8	49.6	38.3	53.6
C33	40.3	43.4	29.7	61.5	31.7	65.8
C34	50.1	55.5	46.2	70.9	46.3	73.9

**Table 7:** Orbit 3D RMS (cm) of different schemes and its improvement.

	Scheme 1	Scheme 2		Scheme 3		Scheme 4	
Satellites	3D RMS	3D RMS	Improvement	3D RMS	Improvement	3D RMS	Improvement
C31	45.2	42.1	6.86%	30.2	33.19%	29.8	34.07%
C32	53.1	49.5	6.79%	34.1	35.78%	31.3	41.05%
C33	137.5	134.2	2.40%	41.6	69.74%	38.1	72.29%
C34	174.1	149.0	14.42%	53.1	69.50%	44.7	74.33%

**Table 8:** The percentage of eliminated data based on BDS-3 orbit determination.

Satellites	Turboedit (Scheme 1)	Improved Algorithm (Scheme 2)
C31	22.14%	8.48%
C32	19.32%	6.42%
C33	43.10%	24.49%
C34	38.96%	18.50%
